# Sulphate of Alumina as an Antiseptic

**Published:** 1843-05-27

**Authors:** 


### SULPHATE OF ALUMINA AS AN ANTISEPTIC.

[We have been handed the subjoined letter for publication. It is additional evidence of the utility of the Sales Aluminæ.—ED. M. EX.]

PUTNAM COUNTY, GEORGIA, May 1st, 1843.

DEAR DOCTOR,—It affords me much satisfaction to see, at your suggestion, the article Sulph. Alumina put to the test, as an antiseptic and detergent, and those properties fully and unequivocally accorded to it by numerous trials in the surgical wards of the Philadelphia Hospital. In corroboration of your views in relation to the antiseptic properties of the Sulph. Alumina, I will merely state to you that I have been using that article as an astringent and antiseptic in fœtid vaginal discharges, (from all causes, except its use was absolutely contra-indicated,) with the happiest effect, for the last six years; it has invariably corrected the fœtor in a short time. It first suggested itself to me as an antiseptic after using it in a case of abortion with retained placenta, succeeded by sanguineo-fœtid discharge, which was very soon corrected by its use as an injection. The strength of the solution has been made in accordance with the sensibility of the parts.

Yours, respectfully,
G. JOHNSON.

Rob'ey Dunglison, M D.

